# Ethical Problems and Moral Distress in Primary Care: A Scoping Review

**DOI:** 10.3390/ijerph18147565

**Published:** 2021-07-16

**Authors:** Noemi Giannetta, Giulia Villa, Federico Pennestrì, Roberta Sala, Roberto Mordacci, Duilio Fiorenzo Manara

**Affiliations:** 1 Faculty of Philosophy, Vita-Salute San Raffaele University, 20132 Milan, Italy; noemigiannetta93@gmail.com (N.G.); pennestri.federico@hsr.it (F.P.); sala.roberta@unisr.it (R.S.); mordacci.roberto@unisr.it (R.M.); 2 Center for Nursing Research and Innovation, Vita-Salute San Raffaele University, 20132 Milan, Italy; manara.duilio@hsr.it

**Keywords:** ethical dilemmas, moral distress, nursing, primary care, community care setting

## Abstract

Background: Since 1997, nursing ethics research has focused on solving ethical dilemmas, enhancing decision-making strategies, and introducing professional education. Few studies describe the triggers of ethical dilemmas among primary care nurses. The aim of this study was to explore the moral distress and ethical dilemmas among primary care nurses. Methods: A scoping review was performed following Arskey and O’Malley’s framework. PubMed, CINAHL, PsycINFO, Embase, and Scopus were searched systematically to retrieve relevant titles and abstracts. A temporal filter was applied to focus on the most recent literature (years of 2010–2020). The research was completed on 17 November 2020. Results: Of 184 articles retrieved, 15 were included in the review. Some (*n* = 7) studies had a qualitative design, and the most productive country was Brazil (*n* = 7). The total number of nurses involved in quantitative studies was 1137 (range: 36–433); the total number of nurses involved in qualitative studies was 144 (range: 7–73). Three main focus areas were identified: (a) frequent ethical conflicts and moral distress episodes among nurses working in primary care settings; (b) frequent moral distress measures here employed; (c) coping strategies here adopted to prevent or manage moral distress. Conclusion: Further research is needed to examine the differences between moral distress triggers and sources of ethical dilemmas among the different care environments, such as primary care and acute care settings.

## 1. Introduction

Many studies have been conducted about the ethical dilemmas faced by nurses in healthcare settings. According to Vošner, Železnik, Kokol, Vošner, and Završnik [1], since 1997, research in the field of nursing ethics has focused on solving ethical dilemmas, enhancing decision making, and introducing education.

In the last two decades, the progress of biomedical science and technology has increased the complexity of the healthcare process [2]. As a consequence, as reported by Haahr, Norlyk, Martinsen, and Dreyer [3], “nurses not only have to take care of needs of patients and their families, [but] they also face multiple demands from medical teams and hospital management in their everyday work”.

During their daily practice, nurses make moral decisions based on their individual awareness and on their own ideas of “good” [4] to understand the best course of action in the interest of the patient [3]. However, “the right thing to do” is sometimes not immediately clear: in many clinical contingencies, the principles of biomedical ethics (autonomy, beneficence, nonmaleficence, and justice) are conflicting, both mutually and against the individual perception of nurses [5].

Based on this, Jameton, in 1984, described three human conditions: moral uncertainty, moral dilemma, and moral distress [6]. Per Jameton’s definition, moral uncertainty is experienced by healthcare workers when they are not aware about what is the right thing to do. Moral dilemmas arise when two or more principles or values conflict and there are mutually inconsistent courses of action. Moral distress arises when “one knows the right thing to do, but institutional constraints make it nearly impossible to pursue the right course of action” [7]. Although these are conflicts for different reasons, the literature tends to describe the resulting episodes of psychological and emotional distress under the common category of moral distress, which is employed uniformly throughout the research to refer to each of the indicated aspects [8].

Several studies have explored the triggers of ethical dilemmas in nursing populations, such as lack of time for care, limited resources, difficult communication with colleagues and other healthcare workers, conflicts with patients and their relatives, and several organizational constraints [8]. With particular reference to the latter, the relationship between moral distress and ethical dilemmas is well-known among nurses, with moral distress understood as the situation in which they are prevented from taking the action they consider morally correct due to environmental pressure and restraints [9,10].

The COVID-19 pandemic faced nurses with further ethical challenges such as admitting patients or not to intensive care units (ICUs), administering or withholding life support, and communicating with their relatives [11,12,13].

During the current pandemic, primary care has received special attention. According to the World Health Organization [14], primary care “is the first contact of people with health services that are continuous, comprehensive and coordinated, has, too often, been focused on treating illness as and when it arises rather than preventing disease in the first place”. Here, nurses play a fundamental role: they practice with considerable independence, and they have a strong and long relationship with patients and their relatives.

Despite the attention received by primary care during the pandemic, the majority of current empiric research is still focused on ethical dilemmas arising in hospital settings [15]. Understanding the ethical dilemmas arising in primary care settings can help nursing leaders and policy makers design strategies to cope with the associated ethical dilemmas and moral distress.

The aim of this study was to explore the moral distress and ethical dilemmas among primary care nurses.

## 2. Materials and Methods

### 2.1. Literature Search

Following Arksey and O’Malley’s framework for scoping reviews [16], we performed a literature review to retrieve evidence about nursing ethical dilemmas and moral distress in primary care settings, and provide a baseline for further research in this area.

The research report of this scoping review was performed according to the PRISMA extension for scoping reviews (PRISMA-ScR; see Supplementary File S1).

This scoping review was conducted to provide knowledge for further research in primary care settings [17]. The following steps were completed: (1) identifying the research questions; (2) identifying relevant studies; (3) selecting studies; (4) extracting collected data; (5) reporting results. The methods and the strategies for conducting these steps are described below.

### 2.2. Step 1: Identify the Research Question

The following research questions were applied in the literature review:

- What ethical dilemmas are experienced by nurses in primary care settings?
- Do nurses experience moral distress in primary care settings?
- How do nurses manage moral distress in primary care settings?
- What are the implications and suggestions for further research?

### 2.3. Step 2: Identifying Relevant Studies

Building on the research questions previously described, we considered three concepts: ethical dilemmas, moral distress, and primary care settings. A search strategy was developed by examining several databases (PubMed, CINAHL, PsycINFO, Embase, and Scopus) for the following keywords: “ethical dilemma”, “moral distress”, “ethical conflict”, “nurses”, “primary care”, and “community care”. The keywords were combined through the Boolean operator OR and AND, and the research was limited to title and abstract. To focus on the most recent literature, a temporal filter was applied (years 2010–2020). The research was completed on 17 November 2020.

### 2.4. Step 3: Selecting Relevant Studies

The screening of studies was performed according to the PRISMA extension for scoping reviews (PRISMA-ScR) [18]. The paper selection was completed by two researchers, after the detection and elimination of duplicate records using Mendeley.

To be included in the synthesis, the studies had to be related to ethical conflict or moral distress in primary care settings and in the nursing population. All the studies that explored the triggers of ethical dilemmas among medical students, nursing students, physicians, psychologists, and all healthcare professions other than nurses were excluded. Studies exploring ethical problems in hospitals, pediatric, and mental or psychiatric settings were also excluded.

### 2.5. Step 4: Extracting Collected Data

All the data were downloaded from the databases on 17 November 2020. The next work stage involved extracting and charting data key information retrieved from the primary research reports. For each potentially relevant study, the following information was collected: author(s), year of publication, title, journal, study design, aims, sample, main measures employed, and main findings (Table 1).

### 2.6. Step 5: Reporting Results

The scoping review results were collated and are reported by themes, consistent with qualitative research protocols.

## 3. Results

### 3.1. Study Selection

**Figure 1:**
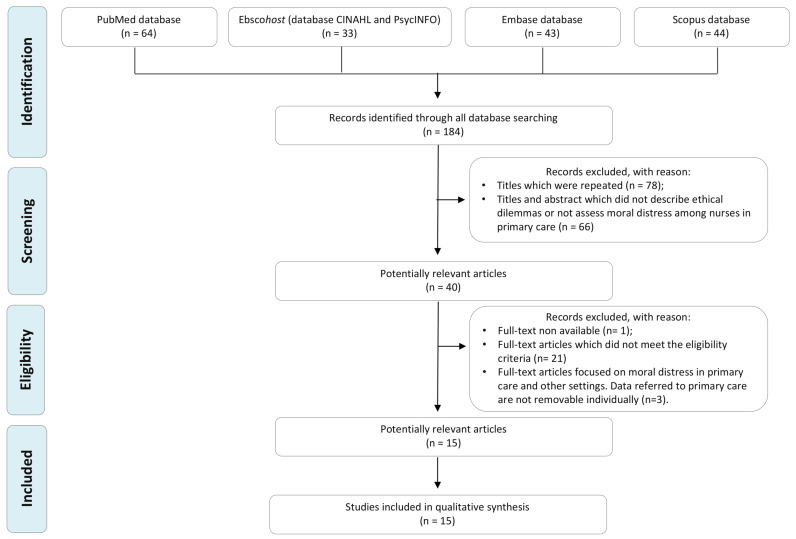
Workflow diagram of the search and selection process, based on the PRISMA flowchart.

The search strategy yielded 184 articles. Of these, 78 were found to be duplicates and were excluded. A further 66 records were excluded after applying eligibility criteria to the title and the abstract. The full texts of the remaining 40 articles were entirely reviewed. Of these, 15 articles met the inclusion criteria and were included in this scoping review. Figure 1 shows the search and selection process, according to the PRISMA statement.

### 3.2. Characteristics of the Studies Included

The majority (*n* = 7) of the studies were qualitative in design [19,20,21,22,23,24,25]: three studies were quantitative [26,27,28], three were validation studies [29,30,31], one study was an integrative review [32], and one was a case study [33].

The most productive country was Brazil, with seven studies included [20,21,25,26,29,30,32]. Two studies were conducted in Canada [24,33]; two in Sweden [22,23]; one each in Germany [19], the Netherlands [28], Turkey [27], and Australia [31]. The total number of nurses involved in quantitative studies was 1137 (range: 36–433); the total number of nurses involved in qualitative studies was 144 (range: 7–73).

### 3.3. Focus Areas

The following three main focus areas were identified: (a) frequent ethical conflicts and moral distress events among primary care nurses; (b) frequent moral distress measures here employed; (c) strategies for moral distress prevention and management in the same setting.

#### 3.3.1. Ethical Conflicts and Moral Distress

Several studies focused on moral distress and the associated triggers experienced by healthcare professionals in primary care settings. Some studies measured its frequency and intensity [28,31]. The majority of them described a low moral distress occurrence, but this experience, when reported, was felt with a moderate level of intensity [31]. de Veer et al. [28] compared the mean of moral distress score in nursing homes, homes for the elderly, home care, and hospitals. Nurses working in nursing homes reported the highest scores, and these scores were significantly higher than those reported by nurses working in home care. In the latter settings, nurses reported the lowest moral distress mean score.

Many factors trigger moral distress in nurses, many of which are related to everyday life, such as poor organization of the working process; conflicting interpersonal relationships among the patient, the community, and the healthcare professionals; controversial management of the ruling system and services provided [25,32]. Barth et al. [25] also identified “lack of qualification of the professional himself”, explained by the contrasting evolution of primary care systems and settings. This is confirmed by findings from Burston et al. [31]. Therefore, nurses need to be provided with new skills to ensure the steady differentiation of people’s health needs and demands.

Gágyor et al. [19], Karlsson et al. [22], and Siqueira-Batista et al. [20] described ethical problems arising in primary care, home care settings, and among family health strategy (FHS) professionals, respectively. According to Karlsson et al. [22], a significant number of the FHS professionals reported no experience with ethical problems during their practice. On the contrary, poor trust from patients or family members, bureaucratic requirements, financial interests from family members, the need to find a balance between the patients’ integrity and protection and between care obligations and care limits, and fearing the negative consequences of certain actions were the main ethical problems reported among primary care nurses [19]. Communication with other healthcare professionals (such as physicians or physiotherapists) was also reported to be poor or conflicting [19,20,22,31]. Nurses “sometimes felt dependent on a family member, a daughter or a son of the patient, who was also involved in patient care” [19]. Primary care nurses reported that they perceived the other parties as a source of ethical problems [19,20]. When the patient’s relatives disagreed with the other healthcare professionals, the situation was even more complicated for nurses. Within community palliative care settings, nurses reported feeling moral distress when they considered a certain patient to need more analgesic treatment but the physician and their colleagues disagreed [23].

Kadıoğlu, Can, Nazik, and Kadıoğlu [27] reported that primary care nurses also encounter ethical issues during their geriatric practice. Most frequently, these issues arise as a part of the decision-making process, with “ignoring respect for privacy during medical procedures and meeting care needs” as the most important worry reported by nurses. These findings are confirmed by Karlsson et al. [23].

Special attention needs to be paid to the ethical problems experienced by community nurses providing end-of-life care at the patient’s own home. Here, nurses reported poor security and uncomfortable feelings when the needs of a dying patient were not satisfied, for instance, when patients in the end-of-life phase deteriorated and required home visits by a physician but the physician did not come. The nurses felt uncomfortable when they did not receive support from the physician at the care center or from the patient’s hospital department when the patient needed more pain relief [22]. Powerlessness, frustration, and concerns were the main feelings experienced by nurses in palliative care [23].

According to Dalla Nora, Campos Pavone Zoboli, and Vieira, [26], moral sensitivity is a precondition to resolving an ethical problem in primary care settings. However, they identified a moderate rate of moral sensitivity among primary care nurses, influenced by interpersonal orientation, professional knowledge, moral conflict, and moral meaning.

#### 3.3.2. Moral Distress Measures in Primary Care Settings

Several instruments are used to assess moral distress in primary care settings. Burston et al. [31] validated the existing instrument, the Moral Distress Scale-Revised, to assess moral distress within the aged-care setting. This instrument, valid and reliable in residential or community aged care, is composed of 20 items, divided into three main factors: quality of care, team capacity, and professional practice. Along the same line, Barth et al. [30] validated an instrument to identify the intensity and frequency of moral distress in primary care. The Brazilian Scale of Moral Distress in Nurses consists of 46 items, divided into six factors: health policies, working conditions, nurse autonomy, professional ethics, disrespect to patient autonomy, and work overload. Based on the findings reported by the authors, this instrument was found to be valid and reliable in primary care. Additionally, de Veer et al. [28] validated an instrument to measure the intensity of moral distress in nursing homes, homes for the elderly, home care, and hospitals.

Recently, Schaefer et al. [29] psychometrically tested the Moral Distress Risk Scale (MDRS) both in acute care settings and in primary care settings. The MDRS consists of a Likert-type scale representing occurrence frequencies of occurrence from one (never) to four (always), each associated with one of the 53 risk factors of moral distress.

#### 3.3.3. Moral Distress Management in Primary Care Settings

Porr et al. [24] developed a theoretical model (Moral Compassing) to explain how community nurses manage challenging ethical situations. Using a qualitative approach, the authors interviewed 24 community nurses, namely, home care and public health nurses. All the participants experienced ethical conflict during the provision nursing care. Based on their story-telling method, Porr et al. [24] described the sequential process that community nurses used when they were faced with an ethical conflict. Firstly, community nurses underwent a visceral reaction when they were aware that “something is not right”: “an instinctive feeling, an uneasiness that nurses describe as a ‘gut feeling’, arises that causes them to pause and consequently their work is disrupted” [24]. This visceral reaction led to the process of self-talk, in which nurses reflected why they “feel a certain way”. Once confident, community nurses sought validation that they were encountering ethical issues by sharing their intuitions with coworkers. This choice helped to increase their confidence in dealing with ethical conflicts and prepared them to manage similar situations through action or inaction. Porr et al., [24] emphasized the importance of coworkers and manager support in helping nurses managing ethical conflicts and/or ethical uncertainty. Several emotions were described by participants during ethical conflict management, such as “angst and upset” that have not “really quite gone away”. Porr et al. [24] described this continuing distress experience as “moral residue”.

Based on a fictitious story, Kayser, Nault, and Ostiguy [33] described three steps to resolve moral distress among registered home care nurses. First, each nurse should focus on the main competing moral principles that cause the distress (promoting beneficence, nonmaleficence, and respecting autonomy). This assessment should be linked to understand the pros and cons of each course of action that can be undertaken. Then, nurses would be able to take action, using the two A’s (ask and act). When applied, nurses should talk with their patients to promote the best solution according to the values and needs of the latter.

The development of ethical competence may be the key to success in resolving ethical conflict and preventing moral distress. In this way, individual values, education, and practice represent a viable path to constructing the process of ethical competence. Organizational and/or educational activities should be promoted as tools for moral distress management, quality of care improvement, and patient safety enhancement in primary care settings [21,32].

## 4. Discussion

With this scoping review, we aimed to describe the main ethical dilemmas and moral distress triggers in primary care settings. Our findings highlight the need to understand moral distress triggers among primary care nurses more in depth as moral distress is a serious problem in nursing, caused mainly by ethical dilemmas.

Many studies have focused on ethical problems and moral distress in hospital settings. Few studies have considered ethical issues in primary care settings. However, on an empirical level, the ethical issues that emerge in the hospital context are generally different from those in primary care settings because the purpose of care is different: in the former case, the goal is to ensure the resolution of acute conditions, aiming for the recovery of the patient; in the latter, the aim is to preserve the best quality of life compatible with the physical, psychological, and social characteristics of the patient. Consequently, at the moral level, the challenges and the strategic–reflexive skills that are used also differ [34,35].

This scoping review identified the following three main focus areas: (a) frequent ethical conflicts and moral distress events among primary care nurses; (b) frequent moral distress measures of moral distress here employed; (c) strategies applied for moral distress prevention and management in the same setting.

The first focus area involves studies exploring the triggers of ethical dilemmas in nursing practice. According to these studies, the frequent triggers of ethical dilemmas are poor organization of the working process, conflicting interpersonal relationships (user, community, and health professionals), and conflicts related to the health service and management system, lack of trust of the patient or family member, bureaucratic requirements, family members’ financial interests, balancing patient integrity and patient protection, care obligations, limits of care, and fearing negative consequences of actions as identified by primary care nurses [19,25,32]. Tense relationships with coworkers are often reported by nurses as a source of moral distress in both hospital care settings [36] and especially in end-of-life care. According to Epstein, Whitehead, Prompahakul, Thacker, and Hamric [37], the three determinants of moral distress are: patient-level, unit/team-level, and system-level.

Even though the literature provides instruments to assess moral distress among healthcare workers [7], few studies measure this experience in primary care settings. The second focus area describes all instruments used in primary care settings to assess moral distress among nurses. Of these, the Moral Distress Scale-Revised aims to measure the intensity and frequency of moral distress [28,31]; differently, the Moral Distress Risk Scale aims to assess the frequency of occurrence for each one of the 53 risk factors for moral distress [29].

The third focus area includes studies describing how to manage and prevent ethical dilemmas and moral distress events. Basically, the development of ethical competence may be the key to success, with nurses being supported by educational activities oriented and tailored to the specific health care setting [38].

The physical, psychological, and social well-being of healthcare workers benefits them and society, which is why investing in their protection is a morally appropriate and economically advantageous choice [39,40]. If moral distress depends not only on individual experiences and choices but also on objective environmental difficulties, intervening in the organization of work represents an interest of the provider (increased safety), the healthcare workers (improved well-being), the patient (higher quality of care), and the society to which they belong (lower expenses related to work absences, operators’ psychophysical recovery, and preventable complications and readmissions) [41].

This scoping review has several limitations. The search strategy was limited to articles written in English or Italian from 2010 to 2020. Therefore, earlier articles or articles written in another language were automatically excluded from this scoping review. Another limitations is that this scoping review did not include quality appraisal of the studies because this kind of review does not formally evaluate the quality of evidence. However, biases may exist in the studies reported. In addition, scoping reviews often gather information from a wide range of study designs and methods, therefore, formal synthesis of the studies and generalization regarding the evidence collected are not provided.

## 5. Conclusions

Ethical dilemmas occur in the everyday practice of primary care nurses. This scoping review supports the need to examine the nursing experience of moral distress in primary care settings in depth to develop more specific organizational and educational strategies. Further research is needed to examine the differences between moral distress triggers and sources of ethical dilemmas among different care environments, such as primary care and acute care settings.

## Figures and Tables

**Table 1:** Summary of findings.

Author(s)	Title	Design of Study	Aim	Sample	Main Measures of MD	Main Findings
Schaefer, R.; Zoboli, E.L.C.P.; Vieira, M.M. (2019)	Psychometric evaluation of the Moral Distress Risk Scale: A methodological study	Validation study	To psychometrically test the Moral Distress Risk Scale.	268 nurses. Of these, 97 working in primary healthcare services.	Moral Distress Risk Scale (MDRS)	“The Moral Distress Risk Scale is composed of 7 factors and 30 items; it shows evidence of acceptable reliability and validity with a Cronbach’s a = 0.913, a total variance explained of 59%, a Kaiser–Meyer–Olkin = 0.896, and a significant Bartlett <0.001”
Porr, C.; Gaudine, A.; Woo, K.; Smith-Young, J.; Green, C. (2019)	How Community Nurses Manage Ethical Conflicts: A Grounded Theory Study	Qualitative study	To uncover the process of behaviors enacted by community nurses when experiencing ethical conflicts	24 community health (as home nurses or public health nurses)	Interviews	“Moral Compassing comprises processes that resolve this main concern by providing community nurses with the means to attain the moral agency necessary to decide to act or to decide not to act. The processes are undergoing a visceral reaction, self-talk, seeking validation, and mobilizing support for action or inaction. [The authors] also discovered that community nurses may experience continuing distress that we labeled moral residue”.
Barth, P.O.; Ramos, F.R.S.; Barlem, E.L.D.; Rennó, H.M.S.; Brehmer, L.C.F.; Rocha, J.M. (2019)	Generating situations of Moral Distress in Primary Care Nurses	Qualitative study	To analyze the situations generating Moral Distress in Primary Care nurses from different regions of Brazil.	13 nurses of primary health care	Semi-structured interviews	Causes of moral distress in primary care settings are related to the professional’s everyday life, such as lack of conditions and organization of the work process, conflicts in interpersonal relationships (user, community, health professionals) and conflicts related to management of services and the health system
Gágyor, I.; Heßling, A.; Heim, S.; Frewer, A.; Nauck, F.; Himmel, W. (2018)	Ethical challenges in primary care: A focus group study with general practitioners, nurses and informal caregivers	Qualitative study	To describe ethical problems from the perspective of these three groups and to investigate whether there is a common experience of ethical issues in primary care.	7 nurses	Focus groups	“Nurses were concerned about bureaucratic and other barriers to professional care and about dual loyalty if they had to consider the conflicting interests of patients and family members. They often felt powerless and unable to act according to their professional standards. Informal caregivers reported problems that resulted from role strain and being both a family member and a caregiver. GPs, nurses and informal caregivers sometimes perceived the other parties as a source of ethical problems”
Barth, P.O.; Ramos, F.R.S.; Barlem, E.L.D.; Dalmolin, G.L.; Schneider, D.G. (2018)	Validation of a moral distress instrument in nurses of primary health care	Validation study	To validate an instrument to identify situations that trigger moral distress in relation to intensity and frequency in primary health care nurses.	433 nurses	Brazilian Scale of Moral Distress in Nurses	“There were 46 questions validated divided into six constructs: Health Policies, Working Conditions, Nurse Autonomy, Professional ethics, Disrespect to patient autonomy and Work Overload. The instrument had satisfactory internal consistency, with Cronbach’s alpha 0.98 for the instrument, and between 0.96 and 0.88 for the constructs.”
Burston, A.; Eley, R.; Parker, D.; Tuckett, A. (2017)	Validation of an instrument to measure moral distress within the Australian residential and community care environments	Validation study	To gain insight into the experience of moral distress within the aged care workforce. To use and validate an existing instrument to measure moral distress within the aged care setting.	106 nurses	Moral Distress Scale- Revised	“The frequency component of the instrument demon- strated an alpha of 0.89, the intensity component 0.95 and the instrument as a whole 0.94. Three factors were identified and labelled as: Quality of Care, Capacity of Team and Professional Practice. Mean scores indicate a low occurrence of moral distress, but this distress, when experienced, was felt with a moderate level of intensity. Primary causes of moral distress were insufficient staff competency levels, poor quality care because of poor communication and delays in implementing palliation.”
Nora, C.R.; Zoboli, E.L.; Vieira, M.M. (2017)	Moral sensitivity in Primary Health Care nurses	Quantitative study	To characterize the profi le and describe the moral sensitivity of primary health care nurses.	100 nurses	Moral Sensitivity Questionnaire	“the nurses had an average moral sensitivity of 4.5 (out of 7). The dimensions with the greatest moral sensitivity were: interpersonal orientation, professional knowledge, moral confl ict and moral meaning.”
Siqueira-Batista, R.; Gomes, A.P.; Motta, L.C.S.; Rennó, L.; Lopes, T.C.; Miyadahira, R.; Vidal, S.V.; Cotta, R.M.M. (2015)	(Bio)ethics and family health strategy: Mapping problems	Qualitative study	To outline the main (bio)ethical problems identified by members of the Family Health Strategy (FHS) teams in the town of Viçosa, Minas Gerais, Brazil.	73 nurses	Interviews	“It was possible to categorize five major groups of (bio)ethical issues experienced by teams: those related to unequal access to health services; those related to the teaching-work-community relation; those related to secrecy and confidentiality; those related to conflicts between team and users; and those related to conflicts between team members.”
Nora, C.R.; Zoboli, E.L.; Vieira, M. (2015)	Ethical problems experienced by nurses in primary health care: integrative literature review	Integrative review	To identify ethical problems experienced by nurses in primary health care and resources for coping based on publications on the subject	-	-	“This analysis resulted in four categories: ethical problems in the relationship between team members, ethical problems in the relationship with the user, ethical problems in health services management and resources for coping with ethical problems. Results showed that nurses need to be prepared to face ethical problems, emphasizing the importance of ethics education during the education process before and during professional practice to enhance the development of ethical sensitivity and competence for problem resolution”
Schaefer, R.; Junges, J.R. (2014)	The construction of ethical competence in the perception of primary care nurses	Qualitative study	To understand the per-ception of nurses of Primary Care Services about the construction of ethical compe-tence on their formation and practices.	10 nurses	Interviews	“The results showed that the interviewed professionals had already experienced situations with ethical conflicts and knew what ethical competen-ce means. The central themes point out three fundamental issues in the construc-tion of the ethical competence: personal values, education and practice. Taking into account that ethical competence is in per-manent construction, the study shows the importance to promote organizational and educational activities in a transversal man-ner, as a tool to cope the moral stress and contribute in improving the quality of care in the primary health attention”
Karlsson, M.; Karlsson, C.; Barbosa da Silva, A.; Berggren, I.; Söderlund, M. (2013)	Community nurses’ experiences of ethical problems in end-of-life care in the patient’s own home	Qualitative study	To gain a deeper understanding of community nurses’ experiences of ethical problems in end-of-life care in the patient′s own home	10 nurses	Inteviews	“In the first step of interpretation, two themes emerged: Uncomfortable feelings and Lack of cooperation and in the second step, one theme Lack of security emerged. Finally, the overall interpretation revealed the theme Feelings of loss of control in end-of-life care in the patient’s own home.”
Kadioǧlu, F.G.; Can, R.; Nazik, S.; Kadioǧlu, S. (2013)	Ethical problems in geriatrics: Views of Turkish primary healthcare professionals	Quantitative study	To determine the frequency rates of various geriatric ethical problems and to evaluate the importance given to these problems in primary healthcare.	36 nurse	Questionnaire	“Based on the results, the most frequently encountered ethical issues were on “decision-making competency” and these issues respectively were “decision-making with relatives instead of elder patients”, “not informing elders due to the lack of tolerance” and “not informing elders due to the lack of comprehending”. The most important geriatric ethical issues were “ignoring respect for privacy”, “ignoring patient’s complaints” and “rejecting detailed examination or treatment because of age”.
De Veer, A.J.E.; Francke, A.L.; Struijs, A.; Willems, D.L. (2013)	Determinants of moral distress in daily nursing practice: A cross sectional correlational questionnaire survey	Quantitative study	To identify individual and job characteristics associated with moral distress in nursing staff	365 nurses	Moral distress questionnaire and MAS-GZ	“Nursing staff in nursing homes had the highest scores and this was statistically significantly higher than those in home care, which had the lowest mean.
Kayser, J.W.; Nault, D.; Ostiguy, G. (2012)	Resolving moral distress when caring for patients who smoke while using home oxygen therapy	Case study	To describe this distress, then to propose a 3-step process of taking concrete actions to resolve the distress	-	-	“Three steps to resolve the moral distress described in this case scenario are proposed. Step 1 entails better understanding the competing moral principles that cause the distress. Step 2 entails better understanding what care options are available for the patient in question. And Step 3 involves taking action.”
Karlsson, M.; Roxberg, A.; Da Silva, A.B.; Berggren, I. (2010)	Community nurses’ experiences of ethical dilemmas in palliative care: A Swedish study	Qualitative study	To highlight community nurses’ experiences of ethical dilemmas in palliative care	7 nurses	Inteviews	“The core themes that emerged were: powerlessness, frustration, and concern in relation to ethical dilemmas in palliative care. The nurses were motivated and felt responsibility for their patients’ end of life, and their relatives, and took their duties seriously. They wanted to satisfy all parties; the patient, the relatives and other palliative care professionals.”

## Data Availability

All data are available upon request.

## Supplementary Materials

The following are available online at https://www.mdpi.com/article/10.3390/ijerph18147565/s1, File S1: PRISMA checklist.

## Abbreviations

ICU: Intensive Care Units; MDRS: Moral Distress Risk Scale; FHS: family health strategy; PRISMA-ScR: Preferred Reporting Items for Systematic reviews and Meta-Analyses extension for. Scoping Reviews.
